# *Tobacco curly shoot virus* Down-Regulated the Expression of nbe-miR167b-3p to Facilitate Its Infection in *Nicotiana benthamiana*

**DOI:** 10.3389/fmicb.2021.791561

**Published:** 2021-12-16

**Authors:** Rui Wu, Gentu Wu, Lyuxin Wang, Xu Wang, Zhuoying Liu, Mingjun Li, Wanzhong Tan, Ling Qing

**Affiliations:** ^1^Chongqing Key Laboratory of Plant Disease Biology, College of Plant Protection, Southwest University, Chongqing, China; ^2^College of Tropical Crops Sciences, Yunnan Agricultural University, Kunming, China

**Keywords:** nbe-miR167b-3p, *Tobacco curly shoot virus*, viral DNA accumulation, miRNA expression, target gene

## Abstract

*Tobacco curly shoot virus* (TbCSV) belongs to the genus *Begomovirus* of the family *Geminiviridae*, and causes leaf curling and curly shoot symptoms in tobacco and tomato crops. MicroRNAs (miRNAs) are pivotal modulators of plant development and host-virus interactions. However, the relationship between TbCSV infection and miRNAs accumulation has not been well investigated. The present study was conducted to analyze different expressions of miRNAs in *Nicotiana benthamiana* in response to the infection of TbCSV via small RNAs sequencing. The results showed that 15 up-regulated miRNAs and 12 down-regulated miRNAs were differentially expressed in TbCSV infected *N. benthamiana*, and nbe-miR167b-3p was down-regulated. To decipher the relationship between nbe-miR167b-3p expression and the accumulations of TbCSV DNA, pCVA mediation of miRNA overexpression and PVX based short tandem target mimic (STTM) were used in this study. It was found that overexpression of nbe-miR167b-3p attenuated leaf curling symptom of TbCSV and decreased viral DNA accumulation, but suppression of nbe-miR167b-3p expression enhanced the symptoms and accumulation of TbCSV. *PRCP*, the target gene of nbe-miR167b-3p, was silenced in plants using VIGS and this weakened the viral symptoms and DNA accumulation of TbCSV in the plants. Overall, this study clarified the effect of nbe-miR167b-3p on plant defense during TbCSV infection, and provided a framework to reveal the molecular mechanisms of miRNAs between plants and viruses.

## Introduction

MicroRNAs (miRNAs) are small RNAs with 20–25 nucleotides that play essential roles in plant biological processes by targeting complementary mRNAs for degradation or translation expression (Bologna and Voinnet, 2014; Cui et al., 2016). In plants, miRNAs regulate leaf morphogenesis, roots and flowers developments, and other key processes (Palatnik et al., 2003; Guo et al., 2005; Zhang et al., 2007). They also function in the response of plants to biotic and abiotic stresses (Wang et al., 2014; Tong et al., 2017), such as inducible expression of miR164 in *Arabidopsis thaliana* led to decreased NAC1 mRNA levels and reduced lateral root emergence (Guo et al., 2005); miRNA guided cleavage of TCP4 mRNA to control the morphogenesis of the leaves (Palatnik et al., 2003); Plants overexpressing osa-miR171b were less susceptible to *Rice stripe virus* (RSV) and virus symptoms were attenuated (Tong et al., 2017); In rice, osa-miR319b played an important role in plant response to cold stress possibly by targeting OsPCF6 and OsTCP21 (Wang et al., 2014).

Viruses cause a variety of symptoms on their host plants, including dwarf, wrinkle, curl, and yellowing, which indicates that plant viruses interfere with the normal growth and development of plants (Maghuly et al., 2014). In recent years, studies have shown that miRNAs participate in the interaction between plants and pathogens (Finnegan and Matzke, 2003; Kidner and Martienssen, 2005; Voinnet, 2005; Du et al., 2011, 2014; Xu et al., 2014; Wang et al., 2016; Zhang et al., 2016; Xia et al., 2019), and viruses change the expression of endogenous miRNAs in plants. For example, RSV enhanced the accumulation of some miRNAs in rice (Du et al., 2011), *Cucumber mosaic virus* (CMV) FNY2b protein suppressed the function of miR159, inducing disease-like symptoms in host plants (Du et al., 2014); The synergistic infection of *Maize chlorotic mottle virus* (MCMV) and *Sugarcane mosaic virus* (SCMV) caused maize lethal necrosis, meanwhile the down-regulation of miR159, miR393, and miR394 was involved in antiviral defense to synergistic infection (Xia et al., 2019). Moreover, different miRNAs participated in anti-virus defense pathways in various plant (Amin et al., 2011). It was also found by our laboratory that different miRNAs can manipulated infected plants to show varied severity of disease symptoms (Du et al., 2020).

It was reported that the suppression of nbe-miR166h-p5 in plants caused leaves to turn dark green with increased chlorophyll, attenuated leaf yellowing symptom of PVX and decreased viral accumulation (Wang et al., 2018). Inhibition of osa-miR171b caused stunting with reduced chlorophyll content in leaves similar to viral symptoms. However, plants overexpressing osa-miR171b were shown less susceptible to RSV and the viral symptoms were attenuated (Tong et al., 2017). When the expression of miR319 was inhibited, the enrichment of endogenous jasmonic acid (JA) was increased in rice, and the resistance of rice was enhanced to *Rice ragged stunt virus* (RRSV) infection (Zhang et al., 2016). These results indicate that miRNAs play an important role in the processes of plant defense and virus infection.

However, the roles and action modes of specific miRNAs involved in viral infection and host susceptibility remain unclear in most cases (Romanel et al., 2012; Gao et al., 2013; Lian et al., 2016). *Tobacco curly shoot virus* (TbCSV) belongs to *Begomovirus*, with a genome of circular single-stranded DNA. TbCSV is transmitted by whitefly (*Bemisia tabaci*) and causes significant losses of tobacco and tomato frequently in China (Li et al., 2005; Xu et al., 2019). Previous studies have shown that inhibiting the expression of nbe-miR1919c-5p can enhance the infection of TbCSV to *N. benthamiana* (Du et al., 2020). At the same time, nbe-miR167b-3p may also respond to the infection of TbCSV (Du et al., 2019). But, there had no experimental evidences to demonstrate the effect of nbe-miR167b-3p on TbCSV infecting host plants. In this study, it was disclosed that the infection of TbCSV could be enhanced by suppressed nbe-miR167b-3p expression and nbe-miR167b-3p responded to the infection of TbCSV by regulating the expression of its target gene *PRCP*.

## Materials and Methods

### Small RNA Library Construction

The leaves of TbCSV inoculated *N. benthamiana* and control plants were collected, and RNA was extracted to construct a small RNA library. Small RNAs sequencing were performed by the Novogene Bioinformatics Technology Company, Beijing, China, using IlluminaHiseq™2500 instrument.

### Vectors Construction

Plasmids for nbe-miR167b-3p expression were constructed with the methods described by Tang et al. (2010): *Cabbage leaf curl virus* (CaLCuV)-based miRNA expression system was developed and the CaLCuV vectors (pCVA and pCVB) used in this study were provided kindly by Dr. Yule Liu of Tsinghua University. The pCVA and pCVB were used for expressing amiRNA of nbe-miR167b-3p. Firstly, the nbe-miR167b-3p and nbe-miR167b-3p* sequences were inserted into Arabidopsis miR319a precursor gene to construct an amiRNA of nbe-miR167b-3p. Then, amiRNA sequence containing two restriction enzyme sites (*Xba*I and *Kpn*I) was synthesized at Beijing Genomics Institute. Next, this amiRNA sequence and pCVA vector were digested with the restriction enzymes *Xba*I and *Kpn*I (TAKARA Bio, 1093S and 1068S) and the amiRNA and pCVA vector were ligated with a ligase (TOYOBO Bio, LGK-101). The recombinated vector pCVA-miR167b-3p was used to overexpress nbe-miR167b-3p.

Virus-based miRNA suppression (VbMS) system was used for miRNA function analysis in *N. benthamiana*. The PVX-miR167b-3p vector was created to mediate nbe-miR167b-3p down-regulation with the technique described and applied by Du et al. (2020). The target mimic was designed to sequester nbe-miR167b-3p using the method of Yan et al. (2014). Then a sequence containing four copies of target mimic and two restriction enzyme sites (*Cla*I and *Sal*I) was synthesized and the copies were separated by a 48 bp nucleotide sequence. This sequence and PVX vector were digested with the restriction enzymes *Cla*I and *Sal*I (TAKARA Bio, 1034S and 1080S), and finally the target mimic of nbe-miR167b-3p and PVX vector were ligated with a ligase (TOYOBO Bio, LGK-101).

By using the psRNA Target tool, the potential target gene of nbe-miR167b-3p was predicted online at http://plantgrn.noble.org/psRNATarget/*Nicotiana benthamiana*, transcript, Niben101 (Dai et al., 2018). The maximum expected value parameter, length of the complementarity score and target accessibility (range 0–100, as small as possible) were set to 2.5, 23 bp and 20.0, respectively. The *Tobacco rattle virus* (TRV)-based virus-induced gene silencing (VIGS) was used to silence target gene of nbe-miR167b-3p in *N. benthamiana* and the target gene was cloned from *N. benthamiana* cDNA. PCR amplification was performed using the HiFi DNA polymerase (TransGen Biotech, Beijing, AP131) and the 300 bp PCR product was gel purified using DNA purification and a recovery kit (TIANGEN Biotech, Beijing, DP201) and combined with the T-vector. The primers used were listed in the Supplementary Table 1. The positive plasmid was digested with the enzymes *Bam*HI and *Sac*I (TAKARA Bio, 1010S and 1078S) and fragment was purified using a DNA purification and recovery kit. Then, it was cloned into the pTRV-RNA2 and digested with the same restriction enzyme site; the recombinant plasmid was transferred into EHA105 strain of *Agrobacterium tumefaciens*.

All of the sequences described above were verified by Sanger sequencing and their sequencing was performed at Beijing Genomics Institute.

### Virus Inoculation and Agrobacterium Infiltration

*N. benthamiana* plants were grown in a greenhouse with settings of 25°C, 16 h of light/day. The virus infectious clones of TbCSV, relative vectors pCVA, pCVB, and pCVA-miR167b-3p, PVX, PVX-miR167b-3p, TRV:GUS, and TRV:PRCP(s) were transformed, respectively, into *A. tumefaciens*. These transformed *A. tumefaciens* were then infiltrated into *N. benthamina* using the treatments described here: (1) *N. benthamiana* plants were infiltrated with the infectious clone of TbCSV at the five-leaf stage and mock-inoculated were used as control. (2) *N. benthamiana* plants were infiltrated with pCVA-miR167b-3p plus pCVB to overexpress nbe-miR167b-3p at the fourth-leaf stage. The plants infiltrated with pCVA plus pCVB were used as control. Symptoms of TbCSV were shown on these plants 11 days post inoculation (dpi). (3) *N. benthamiana* plants were infiltrated with PVX-miR167b-3p to down-regulate the expression of nbe-miR167b-3p at the five-leaf stage and PVX inoculated plants were used as control. At 7 dpi, these plants were infiltrated with TbCSV. (4) For co-infection, equal concentrations and volumes of individual *A. tumefaciens* cultures were mixed. Twenty plants were used for each treatment and the control. The symptoms on diseased plants were observed and photographed. The leaves at the same position were harvested, and virus accumulation and gene expression in the leaves were further detected with qPCR and qRT-PCR.

### DNA Extraction and Virus Accumulation Detection

The DNAs of *N. benthamiana* were extracted using the cetyl trimethylammonium bromide (CTAB) method. To determine whether *N. benthamiana* plants were infected by TbCSV, specific primers of TbCSV were used to amplify the virus DNA in the diseased leaves with PCR. To detect virus accumulation in the leaves, the qRCR technique (Zorzatto et al., 2015) was applied and the result of qRCR was calculated according to the absolute method (Schmittgen and Livak, 2008; Rodríguez-Negrete et al., 2014). The 20 μL reaction solution system of qPCR contained 10 μL NovoStart^®^ SYBR qPCR Super Mix Plus, 8 μL RNase free water, 0.5 μL AV1-qF (10 μM), 0.5 μL AV1-qR (10 μM), and 50 ng DNA template. The linear standard curve of TbCSV was automatically generated by Origin 9.0 software based on the lg (log10) value of the copy number of TbCSV in each sample. Each qPCR reaction was repeated three times (batches) with 24 plants of *N. benthamiana* in each repeated batch.

### Quantitative Reverse Transcriptase PCR Analysis

The total RNA of *N. benthamiana* was extracted using TRIzol reagent (Invitrogen, California, United States). The qRT-PCR for nbe-miR167b-3p was based on previous reports (Guo et al., 2012). The specific RT stem-loop primers in the Prime Script RT reagent Kit (TAKARA Bio, Kusatsu, Shiga, Japan) were used to reverse the RNA and the final reverse transcribed products from qRT-PCR was analyzed using the SYBR green kit supplied by Novoprotein, Shanghai, China. The qRT-PCR parameters were set as 95°C pre-denaturation 3 min, 32 cycles of 95°C denaturation 25 s and 60°C annealing 30 s, and a final extension at 72°C for 30 s, using the action gene (XM_033660572.1) of *N. benthamiana* as the internal reference. The 2^–ΔΔCT^ method (Du et al., 2020) was used for qRT-PCR analysis. For statistical analysis, three fully independent biological replicates were designed and subjected to qRT-PCR tests.

For detecting expression level of the target gene, NovoStart ®SYBR qPCR Super Mix Plus kit (NovoProtein) was employed to perform qRT-PCR on the CFX 96 real-time system (Bio-Rad). Three biological samples for each qRT-PCR reaction were processed and each biological sample was repeated three times. The experimental results were computed from data of three biological repeats.

## Results

### Effect of *Tobacco curly shoot virus* on the Expression of MicroRNAs in *Nicotiana benthamiana*

**FIGURE 1:**
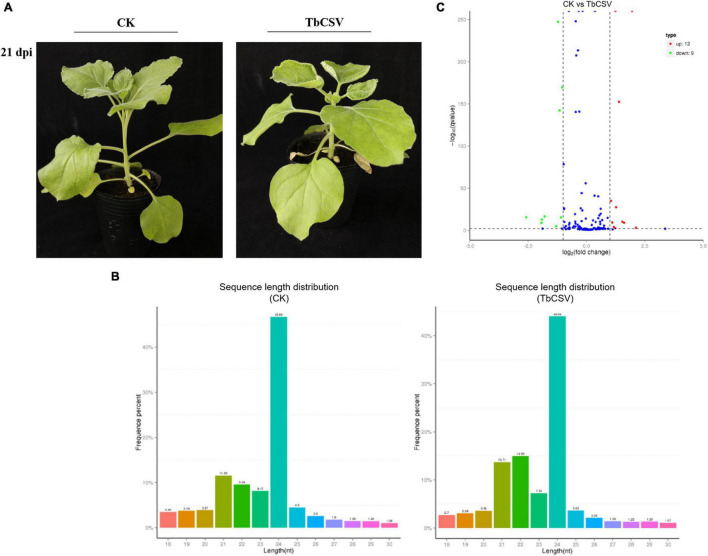
Analysis of small RNA sequencing data. **(A)** TbCSV symptoms on systemic leaves of *N. benthamiana* plants at 21 dpi with leaf curling and shrinking. **(B)** The length distribution of small RNAs after TbCSV infecting *N. benthamiana*. The abscissa shows the size of miRNAs and vertical axis shows percentage of total small RNAs. **(C)** Volcano map of differential expression miRNAs in TbCSV infiltrated *N. benthamiana*. The abscissa shows the fold change difference in the expression of miRNAs in different treatments and control plants, and the vertical axis indicates the adjusted p-adj for the differences in expression. MiRNAs without significant differences are indicated by blue dots, the up-regulated genes by red dots and the down-regulated genes by green dots.

Typical disease symptoms with leaf curling and shrinking developed on inoculated *N. benthamiana* plants 21 days after TbCSV inoculation while the control plants remained symptomless and healthy (Figure 1A). Through sequencing analysis of the small RNAs from leaves collected at 21 dpi, 5,982,433 and 5,675,863 specific small RNA sequences were detected in, and a small RNA library was constructed for, the inoculated and healthy plants, respectively. Of the unique reads, 4,762,789 (45.63%) were from the control plants and 4,456,219 (42.69%) from the inoculated plants (Table 1). Statistical analysis of the data showed that the content of 21nt sRNA was more than that of 22 nt sRNA in control plants, while in TbCSV-infected samples, the content of 21nt sRNA was less than the content of 22 nt sRNA in TbCSV-infected plants (Figure 1B). Compared with miRNA in control plants, there were 21 miRNAs expressed differentially in TbCSV-infected plants, including 12 up-regulated and 9 down-regulated miRNAs (Figure 1C).

**TABLE 1:** Distribution of small RNA sequences among the two constructed libraries.

Small RNA library	Specific sRNAs reads	Unique sRNAs reads	% in specific
CK	5,982,433	4,762,789	45.63%
TbCSV	5,675,863	4,456,219	42.69%

### *Tobacco curly shoot virus* Induced Leaves Curling Symptom and Down-Regulated Expression of nbe-miR167b-3p

**FIGURE 2:**
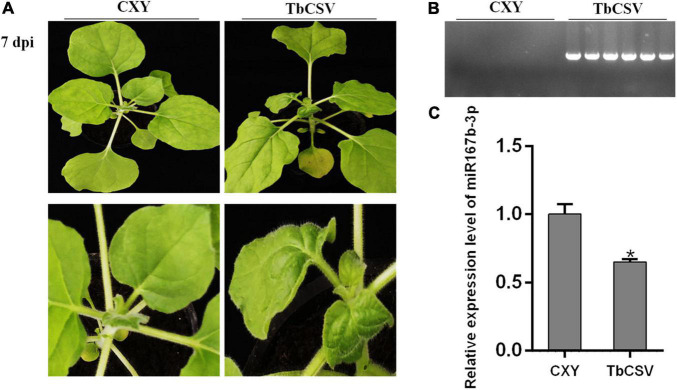
TbCSV symptoms at 7 dpi and differential expression of nbe-miR167b-3p in TbCSV-infected *N. benthamiana*. **(A)** Leaf curling symptom caused by TbCSV in *N. benthamiana* leaves. **(B)** Analysis of TbCSV DNA through PCR. **(C)** Quantitative RT-PCR demonstrating the expression of nbe-miR167b-3p in TbCSV-infected in *N. benthamiana* plants. Significance of difference (*p < 0.05) was between the treatments was determined by the Student’s *t*-test.

Significant and typical systematic curling symptom developed on leaves of *N. benthamiana* plants, at 7 dpi with the infectious clone of TbCSV, but no symptom was observed on the control plant (Figure 2A). PCR tests showed that the DNA of TbCSV existed in the leaves showing symptoms but not in the control plants (Figure 2B).

According to the results of small RNA sequencing, it was found that TbCSV infection down-regulated the expression of nbe-miR167b-3p in *N. benthamiana*. qRT-PCR tests illustrated that the expression level of nbe-miR167b-3p in the infected *N. benthamiana* plants was down-regulated to about 65%, compared with that in the control plant leaves (Figure 2C).

### Silencing nbe-miR167b-3p Expression Aggravated Disease Symptoms and DNA Accumulation of *Tobacco curly shoot virus*

**FIGURE 3:**
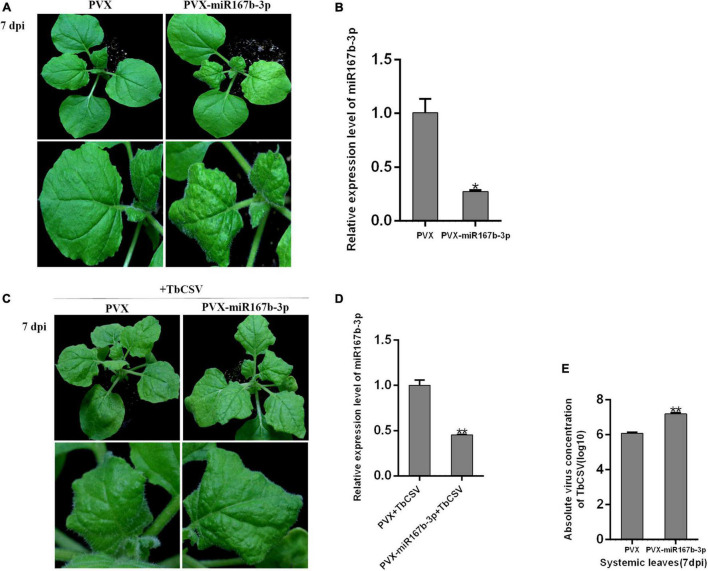
Suppression of nbe-miR167b-3p enhanced leaf curling symptom induced by TbCSV and increased viral DNA accumulation. **(A)** PVX-based miRNA suppression (VbMS) system was used to down-regulate the expression of nbe-miR167b-3p. **(B)** Expression level of nbe-miR167b-3p in PVX-miR167b-3p-treated plants was significantly reduced compared with that in the control plants by qRT-PCR. **(C)** TbCSV was infiltrated into PVX-miR167b-3p-treated and control plants 7 days after VbMS treatment. **(D)** Relative expression level of nbe-miR167b-3p in TbCSV-infected and PVX-miR167b-3p-treated plants using qRT-PCR. **(E)** TbCSV DNA copy numbers in the systemic leaves harvested from the PVX plus TbCSV-inoculated plants or the PVX-miR167b-3p plus TbCSV-inoculated plants. Significance of difference between treatments (*p < 0.05; **p < 0.01) was determined by the Student’s *t*-test.

For exploring specific function of nbe-miR167b-3p in TbCSV infecting *N. benthamiana*, a vector was constructed to silence nbe-miR167b-3p (PVX-miR167b-3p) using *Potato virus X* (PVX) infectious clone. Then, *A. tumeficiens* GV3101 transformed with PVX and PVX-miR167b-3p was resuspended and the suspensions (OD_600_ = 1.0) were infiltrated into *N. benthamiana*, respectively. At 7 dpi, the upper leaves showed significant curling symptom on the PVX-miR167b-3p treated plants, while only the mosaic symptom was observed on leaves of control plants (Figure 3A). Compared with the PVX-inoculated plants, the expression level of nbe-miR167b-3p in the PVX-miR167b-3p was significantly suppressed and the expression was down-regulated to only 27.4% of that in the control plants (Figure 3B).

When inoculated with TbCSV by agroinfiltration, all plants of the above two treatments showed leaf curling symptom at 7 dpi, and the symptom on the PVX-miR167b-3p plus TbCSV-inoculated plants were more severe than that on the control (PVX plus TbCSV) plants (Figure 3C). Results from qRT-PCR analysis showed that the relative expression level of nbe-miR167b-3p in the plants inoculated with PVX-miR167b-3p plus TbCSV was down-regulated significantly, compared with that in the plants inoculated with PVX plus TbCSV (Figure 3D). Detection of DNA accumulation with qPCR at 7 dpi showed that the amount of viral DNA in the systematically infected leaves of the plants inoculated with PVX-miR167b-3p plus TbCSV treatment was higher than that of the plants inoculated with PVX plus TbCSV (Figure 3E). These results suggested that silencing of nbe-miR167b-3p in *N. benthamiana* was beneficial to TbCSV infection.

### Overexpression of nbe-miR167b-3p Alleviated Symptoms and *Tobacco curly shoot virus* DNA Accumulation

**FIGURE 4:**
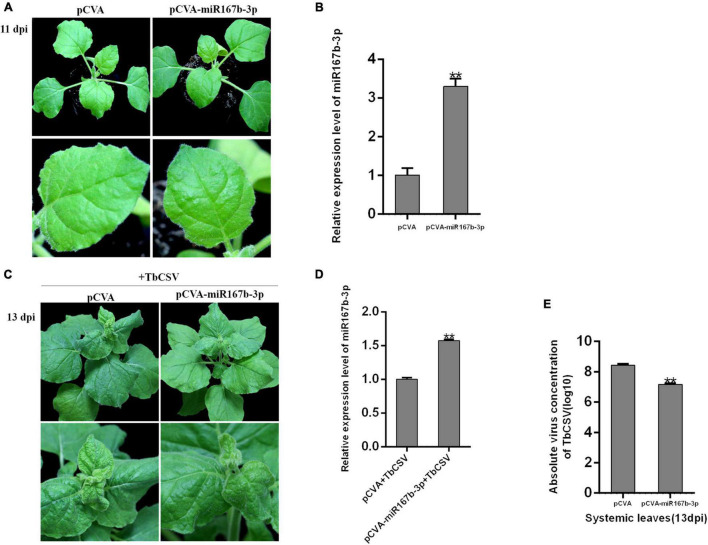
Overexpression of nbe-miR167b-3p attenuated leaf curling symptom induced by TbCSV and reduced viral DNA accumulation. **(A)** pCVA-based miRNA vector (pCVA-miR167b-3p) was used to increase expression of nbe-miR167b-3p. **(B)** Result of qRT-PCR showed that expression of nbe-miR167b-3p in the pCVA-miR167b-3p-inoculated plants was significantly increased compared with that of the control plants (pCVA). **(C)** TbCSV was inoculated onto pCVA-miR167b-3p -treated and control plants. **(D)** qRT-PCR showed that the level of nbe-miR167b-3p in TbCSV-infected and pCVA-miR167b-3p-treated plants was significantly increased compared with that of the TbCSV-infected pCVA-treated plants. **(E)** TbCSV DNA copy numbers in the systemic leaves harvested from the pCVA plus TbCSV-inoculated plants or the pCVA-miR167b-3p plus TbCSV-inoculated plants. Significance of difference between treatments (*p < 0.05; **p < 0.01) was determined by the Student’s *t*-test.

CaLCuV (pCVA) vector was used to investigate the effect of nbe-miR167b-3p up-expression on TbCSV infection of *N. benthamiana*. Through DNA recombination, pCVA-miR167b-3p plasmid was constructed and transformed into *A*. *tumeficiens* EHA105 strain. Nbe-miR167b-3p was expressed in *N. benthamiana* plants with pCVA-miR167b-3p plus pCVB through agroinfiltration and the control plants were inoculated with pCVA plus pCVB. The expression level of nbe-miR167b-3p was detected in the upper leaves at 11 dpi. The result showed that there was no significant difference in symptoms between the treated and the control plants (Figure 4A). However, compared with that in the pCVA plus pCVB-inoculated plants, the expression level of nbe-miR167b-3p in the pCVA-miR167b-3p plus pCVB-inoculated plants was up-regulated significantly and this overexpression was 3.3 times of that in the control plants (Figure 4B).

All plants inoculated with pCVA plus pCVB or pCVA-miR167b-3p plus pCVB were infiltrated at 11 dpi with TbCSV by agroinfiltration. At 13 dpi, the upper leaves in both of the treated and control plants showed upward leaf curling symptom, and the symptom in pCVA-miR167b-3p plus pCVB and TbCSV plants was less severe than that on the control plants (Figure 4C). At this stage, qRT-PCR showed that the relative expression level of nbe-miR167b-3p in the plants inoculated with pCVA-miR167b-3p plus pCVB and TbCSV was still up-regulated significantly, compared with that in the control plants (Figure 4D). Leaves of the plants were harvested to detect the accumulation of TbCSV DNA with qPCR and the results showed that the accumulation of TbCSV DNA in the leaves of pCVA-miR167b-3p plus pCVB and TbCSV infiltrated plants was significantly lower than that in the control plant leaves (Figure 4E). These results indicated that overexpression of nbe-miR167b-3p in *N. benthamiana* inhibited TbCSV infection.

### The Expression of nbe-miR167b-3p Target Gene

**FIGURE 5:**
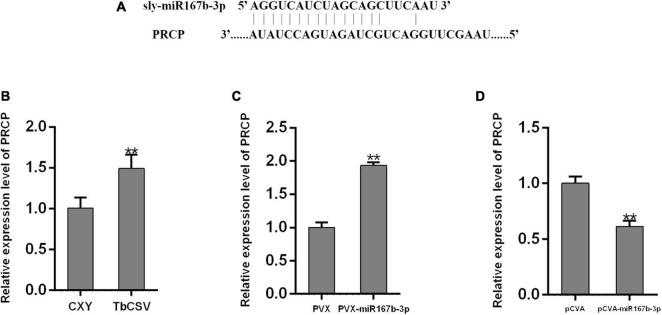
Analyses of the expression of nbe-miR167b-3p target gene *PRCP* through qRT-PCR. **(A)** The binding site of nbe-miR167b-3p on the mRNAs of potential target was localized at the encoding sequence. **(B)** The mRNA levels of *PRCP* were up-regulated in TbCSV-infected *N. benthamiana* plants. **(C)** The mRNA levels of *PRCP* were up-regulated in nbe-miR167b-3p-suppressed leaves. **(D)** The mRNA levels of *PRCP* were down-regulated in nbe-miR167b-3p-overexpressed leaves. Significance of difference between treatments (*p < 0.05; **p < 0.01) was determined by the Student’s *t*-test.

To explore how nbe-miR167b-3p regulates the infection of TbCSV in *N. benthamiana*, the potential target genes were analyzed using psRNA Target online at http://plantgrn.noble.org/psRNATarget/ and the results were given in Supplementary Table 2. Specific primers were designed with the sequences of target genes and qRT-PCR was performed to detect the expression level of these genes. The results showed that, compared with the mock-inoculated plants (CXY), the expression level of the target gene pentatricopeptide repeat-containing protein (PCRP) (Figure 5A) in the TbCSV-infected plants was significantly up-regulated (Figure 5B). This indicated that the expression level of *PRCP* was up-regulated significantly in the PVX-miR167b-3p infiltrated plants; this was 1.9 times of that in the PVX-inoculated plants (Figure 5C). Furthermore, the relative expression of *PRCP* in the pCVA-miR167b-3p plus pCVB inoculated plants was also detected by qRT-PCR and the results showed that the expression of *PRCP* was down-regulated and this was 61.4% of that in the control plants (Figure 5D).

### Silencing of *PRCP* Reduced Infection of *Tobacco curly shoot virus* in *Nicotiana benthamiana*

**FIGURE 6:**
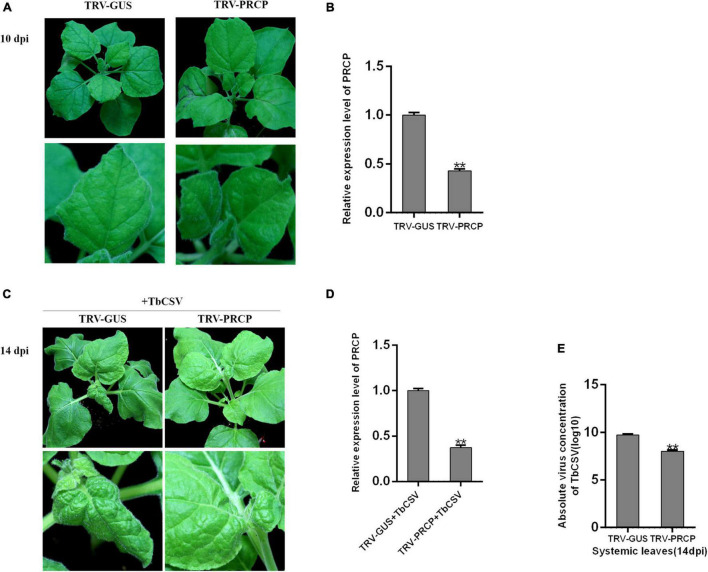
Effect of *PRCP* on TbCSV pathogenicity. **(A)** *Tobacco rattle virus* (TRV)-based vector (TRV-PRCP) was used to down-regulate the expression of *PRCP*. **(B)** The expression level of *PRCP* in TRV-PRCP-treated plants was significantly reduced compared with that in control plants. **(C)** TbCSV was inoculated onto TRV-PRCP-treated and control plants 10 days after VIGS treatment. **(D)** The level of *PRCP* in TbCSV-infected and TRV-PRCP-treated plants was significantly down-regulated compared with that in TbCSV-infected and TRV-GUS-treated plants. **(E)** Detections of TbCSV DNA copy numbers in the systemic leaves harvested from the TRV-GUS plus TbCSV-inoculated plants or the TRV-PRCP plus TbCSV-inoculated plants. Significance of difference between treatments (*p < 0.05; **p < 0.01) was determined by the Student’s *t*-test.

To investigate the function of nbe-miR167b-3p target gene *PRCP* in the process of TbCSV infection, *Tobacco rattle virus* (TRV)-induced VIGS was used and recombinant vector (TRV-PRCP) was created to silence *PRCP* in *N. benthamiana*. TRV-PRCP was transformed into *A*. *tumeficiens* EHA105 and 12 *N. benthamiana* plants were inoculated through the transformants; the control plants were inoculated with TRV-GUS. At 10 dpi, there was not significantly difference of the growth and development between TRV-PRCP-infiltrated and control plants (Figure 6A). qRT-PCR tests showed that the expression of *PRCP* in the TRV-PRCP infected plants was significantly decreased, to 43% of the expression level in the control plants (Figure 6B).

When all the plants were inoculated with TbCSV, the *N. benthamiana* plants inoculated with TRV-PRCP plus TbCSV developed little leaf curling symptom, which was similar to the leaf curling symptom induced by TbCSV. In contrast, severe leaf curling symptom was observed on the control plants (Figure 6C). The result of qRT-PCR illustrated that the expression of *PRCP* was suppressed significantly in the TRV-PRCP plus TbCSV-inoculated plants, compared with that in the control plants (Figure 6D). qPCR detection also found that the viral DNA accumulation in TRV-PRCP plus TbCSV-inoculated plants was down-regulated compared with that in the control plants (Figure 6E). These results suggested that silencing of nbe-miR167b-3p target gene *PRCP* in *N. benthamiana* inhibited the TbCSV infection.

## Discussion

A number of studies reported that miRNAs played important roles in plant growth and resistance to pathogen infections. It was verified that miRNA-triggered changes in gene expression were essential for controlling plant development and for modulating the adaptation of plants to pathogen infection and the infection by different viruses changed the level of certain miRNAs in plants. For example, studies of Yang et al. (2016) showed that 56 miRNAs were up-regulated and 13 miRNAs were down-regulated by RSV infection, providing new insights into the mechanisms of RSV pathogenicity. In transgenic *A. thaliana* plants, expression of *Cucumber mosaic virus* (CMV) 2b silencing suppressor protein from the severe strain Fny of subgroup IA disrupted miRNA159a-regulated development, which caused severe symptoms on the plants (Du et al., 2014). After infection of Chinese cabbage by *Turnip mosaic virus* (TuMV), the expression of bra-miR1885 and bra-miR158 was significantly up-regulated (He et al., 2008). The change of microRNA expression was considered as the main factor inducing symptoms after virus infection (Yang et al., 2016). Meanwhile, some changes in the expression level of miRNAs may also lead to leaf yellowing, curling, wrinkling, and dwarfing. In our previous experiment, different expressions of miRNAs in *N. benthamiana* during the TbCSV infection were analyzed (Du et al., 2019), but only limited examples of specific virus-inducible miRNAs that were directly involved in viral infection or host susceptibility existed (Zhang et al., 2016). Results from the present study showed that nbe-miR167b-3p expression was suppressed by TbCSV infection and that nbe-miR167b-3p target gene *PRCP* changed plant growth and susceptibility of *N. benthamian* to TbCSV.

Some studies indicated that miR167 was involved in plant development, biostress and abiostress. MiR167a was shown to affect the expression of auxin response factors (ARF6 and ARF8) and to induce flower defection and female sterility in tomato (Liu et al., 2014). MiR167 also induced certain changes in leaf shape, stomatal closure and relative water content by regulating differential expressions of its target genes *MesARF6* and *MesARF8*, thus resulting in response to cassava water deficiency (Phookaew et al., 2014). MiR167b inhibited the infection of PVY and PVX by targeting RNA silencing suppressor HC-Pro of PVY and the TGBp1/p25 (p25) of PVX; this improved tobacco resistance to PVY and PVX (Ai et al., 2011). In our study, the expression level of nbe-miR167b-3p was down-regulated in TbCSV-infected plants. Because nbe-miR167b-3p was inhibited in the process of TbCSV infection, it was speculated that nbe-miR167b-3p played an active role in resistance of *N. benthamiana* to TbCSV infection.

RRSV infection was indicated to increase the accumulation of miR319 in rice plants and simultaneously suppressed the JA-mediated defense against virus infection and symptom development (Zhang et al., 2016). The studies of Wu et al. (2015) revealed that the antiviral function of AGO18 depended on its activity to sequester miR168 to alleviate repression of rice; AGO1 essential for antiviral RNAi increased rice antiviral activity miR528 and negatively regulated viral resistance in rice by cleaving L-ascorbate oxidase (AO) messenger RNA, and thereby reduced AO-mediated accumulation of reactive oxygen. Upon viral infection, AGO18 isolated miR528 leading to elevated AO activity, higher basal reactive oxygen accumulation and enhanced antiviral defense (Wu et al., 2017). In the present study, nbe-miR167b-3p was found to down-regulate the expression by PVX-induced gene silencing system, and these caused downward leaf curling on *N. benthamiana* plants. The silencing of nbe-miR167b-3p aggravated the symptoms of TbCSV infection and increased the accumulation of TbCSV DNA in *N. benthamiana*. Then an expression vector overexpressing nbe-miR167b-3p (pCVA-miR167b-3p) was constructed using the pCVA vector and after inoculating *N. benthamiana* plants with this expression vector, it was found that the up-regulated expression of nbe-miR167b-3p in *N. benthamiana* not only alleviated symptoms of TbCSV infection, but also reduced the accumulation of TbCSV in *N. benthamiana* plants.

Experimental results of our study showed that the change in expression level of the target gene *PRCP* predicted by nbe-miR167b-3p was regulated by TbCSV infection. The gene mainly encodes a PRCP. PRCP proteins are defined by tandem repeats of a degenerated 35 amino acid motif (Small and Peeters, 2000). The PRCP family is one of the largest protein families in plants and there are 450 and 477 PRCP proteins in *Arabidopsis* and rice, respectively. PRCP proteins were found to play a central and broad role in modulating the expression of organelle genes. For example, delayed greening1 (*DG1*) and yellow seedling1 (*YS1*) mutants in *Arabidopsis* displayed seedling-stage-specific albino and yellow seedling phenotypes, respectively. Both *DG1* and *YS1* encode chloroplast-targeted PRCP proteins (Zhou et al., 2009; Chi et al., 2010). However, the functions of the *PRCP* gene in plant responses to virus infection still remain unknown. In this study, it was found that inhibiting expression of nbe-miR167b-3p in plants induced the up-regulation of *PRCP*, while overexpression of nbe-miR167b-3p induced *PRCP* down-regulation. Further, the knockdown of *PRCP* gene did not affect normal growth of tobacco plants, but reduced TbCSV DNA accumulation in and disease symptom development on the plants. In summary, it was demonstrated that TbCSV infection in *N. benthamiana* reduced the expression of nbe-miR167b-3p, which targeted *PRCP* genes to regulate plant development and enhanced defense of the plants against virus infection. The study did not determine the mechanism by which expression of nbe-miR167b-3p was down-regulated by TbCSV infection, or how *PRCP* affected the pathogenic mechanism of TbCSV. Further studies are necessary to examine and illustrate these mechanisms.

## Conclusion

To conclude, results from this study showed that TbCSV down-regulated the expression of nbe-miR167b-3p in *N. benthamiana*. The silencing of nbe-miR167b-3p not only affected normal development of *N. benthamiana* plants and induced downward leaf curling, but also aggravated the viral symptoms and increased the accumulation of TbCSV in *N. benthamiana* plants. On the contrary, the overexpression of nbe-miR167b-3p alleviated the symptoms of TbCSV, and reduced the accumulation of TbCSV in *N. benthamiana*. It was also proved that nbe-miR167b-3p responded to the infection of TbCSV by regulating the expression of its target gene *PRCP*. So far, there has been no report on the action of nbe-miR167b-3p on TbCSV infection.

## Data Availability Statement

The original contributions presented in the study are included in the article/Supplementary Material, further inquiries can be directed to the corresponding author/s.

## Author Contributions

RW and GW performed the vector constructions and the main experiments, data processing and analysis, and developed the research program. LW participated small RNA library construction. XW and ZL carried out the sampling and the sample processing. ML participated throughout the investigations. WT advised on the research program and revised the manuscript. LQ conceived the study and revised the manuscript. All authors contributed to the article and approved the submitted version.

## Conflict of Interest

The authors declare that the research was conducted in the absence of any commercial or financial relationships that could be construed as a potential conflict of interest.

## Publisher’s Note

All claims expressed in this article are solely those of the authors and do not necessarily represent those of their affiliated organizations, or those of the publisher, the editors and the reviewers. Any product that may be evaluated in this article, or claim that may be made by its manufacturer, is not guaranteed or endorsed by the publisher.

## References

[B1] Ai T. Zhang L. Gao Z. Zhu C. X. Guo X. (2011). Highly efficient virus resistance mediated by artificial microRNAs that target the suppressor of PVX and PVY in plants. *Plant Biol. (Stuttg)* 13 304–316. 10.1111/j.1438-8677.2010.00374.x 21309977

[B2] Amin I. Patil B. L. Briddon R. W. Mansoor S. Fauquet C. M. (2011). A common set of developmental miRNAs are upregulated in *Nicotiana benthamiana* by diverse Begomoviruses. *Virol. J.* 8 143. 10.1186/1743-422X-8-143 21447165 PMC3072929

[B3] Bologna N. G. Voinnet O. (2014). The diversity, biogenesis, and activities of endogenous silencing small RNAs in Arabidopsis. *Annu. Rev. Plant Biol.* 65 473–503. 10.1146/annurev-arplant-050213-035728 24579988

[B4] Chi W. Mao J. Li Q. Ji D. Zou M. Lu C. (2010). Interaction of the pentatricopeptide-repeat protein delayed greening 1 with sigma factor SIG6 in the regulation of chloroplast gene expression in *Arabidopsis* cotyledons. *Plant J.* 64 14–25. 10.1111/j.1365-313X.2010.04304.x 20626654

[B5] Cui J. You C. Chen X. (2016). The evolution of microRNAs in plants. *Curr. Opin. Plant Biol.* 35 61–67. 10.1016/j.pbi.2016.11.006 27886593 PMC5342909

[B6] Dai X. Zhuang Z. Zhao P. X. (2018). PsRNATarget: a plant small RNA target analysis server. *Nucleic Acids Res.* 46 W49–W54. 10.1093/nar/gky316 29718424 PMC6030838

[B7] Du J. Wu G. Zhou Z. Zhang J. Li M. Sun M. (2019). Identification of microRNAs regulated by *Tobacco curly shoot virus* co-infection with its betasatellite in *Nicotiana benthamiana*. *Virol. J.* 16:130. 10.1186/s12985-019-1234-5 31699111 PMC6836351

[B8] Du J. Wu R. Liu Z. Sun M. Ghanem H. Li M. (2020). Suppression of nbe-miR1919c-5p expression in *Nicotiana benthamiana* enhances *Tobacco curly shoot virus* and its beta satellite co-infection. *Viruses* 12:392. 10.3390/v12040392 32244650 PMC7232422

[B9] Du P. Wu J. Zhang J. Zhao S. Zheng H. Gao G. (2011). Viral infection induces expression of novel phased microRNAs from conserved cellular microRNA precursors. *PLoS Pathog.* 7:e1002176. 10.1371/journal.ppat.1002176 21901091 PMC3161970

[B10] Du Z. Chen A. Chen W. Westwood J. H. Baulcombe D. C. Carr J. P. (2014). Using a viral vector to reveal the role of microRNA159 in disease symptom induction by a severe strain of *Cucumber mosaic virus*. *Plant Physiol.* 164 1378–1388. 10.1104/pp.113.232090 24492335 PMC3938627

[B11] Finnegan E. J. Matzke M. A. (2003). The small RNA world. *J. Cell Sci.* 116 4689–4693. 10.1242/jcs.00838 14600255

[B12] Gao R. Wan Z. Y. Wong S. M. (2013). Plant growth retardation and conserved miRNAs are correlated to Hibiscus chlorotic ringspot virus infection. *PLoS One* 8:e85476. 10.1371/journal.pone.0085476 24386476 PMC3875576

[B13] Guo H. S. Xie Q. Fei J. F. Chua N. H. (2005). MicroRNA directs mRNA cleavage of the transcription factor NAC1 to downregulate auxin signals for Arabidopsis lateral root development. *Plant Cell* 17 1376–1386. 10.1105/tpc.105.030841 15829603 PMC1091761

[B14] Guo W. Wu G. Yan F. Lu Y. Zheng H. Lin L. (2012). Identification of novel *Oryza sativa* miRNAs in deep sequencing-based small RNA libraries of rice infected with *Rice stripe virus*. *PLoS One* 7:e46443. 10.1371/journal.pone.0046443 23071571 PMC3468594

[B15] He X. F. Fang Y. Y. Feng L. Guo H. S. (2008). Characterization of conserved and novel microRNAs and their targets, including a TuMV-induced TIR-NBS-LRR class R gene-derived novel miRNA in *Brassica*. *FEBS. Lett.* 582 2445–2452. 10.1016/j.febslet.2008.06.011 18558089

[B16] Kidner C. A. Martienssen R. A. (2005). The developmental role of microRNA in plants. *Curr. Opin. Plant Biol.* 8 38–44. 10.1016/j.pbi 15653398

[B17] Li Z. Xie Y. Zhou X. (2005). *Tobacco curly shoot virus* DNA beta is not necessary for infection but intensifies symptoms in a host-dependent manner. *Phytopathology* 95 902–908. 10.1094/PHYTO-95-0902 18944412

[B18] Lian S. Cho W. K. Kim S. M. Choi H. Kim K. H. (2016). Time-ocurse small RNA profiling reveals rice miRNAs and their target genes in response to *Rice stripe virus* infection. *PLoS One* 11:e0162319. 10.1371/journal.pone.0162319 27626631 PMC5023111

[B19] Liu N. Wu S. Van, Houten J. Wang Y. Ding B. (2014). Down-regulation of auxin response factors 6 and 8 by microRNA167 leads to floral development defects and female sterility in tomato. *J. Exp. Bot.* 65 2507–2520. 10.1093/jxb/eru141 24723401 PMC4036516

[B20] Maghuly F. Ramkat R. C. Laimer M. (2014). Virus versus host plant microRNAs: who determines the outcome of the interaction? *PLoS One* 4:e98263. 10.1371/journal.pone.0098263 24896088 PMC4045720

[B21] Palatnik J. F. Allen E. Wu X. Schommer C. Schwab R. Carrington J. C. (2003). Control of leaf morphogenesis by microRNAs. *Nature* 425 257–263. 10.1038/nature01958 12931144

[B22] Phookaew P. Netrphan S. Sojikul P. Narangajavana J. (2014). Involvement of miR164- and miR167-mediated target gene expressions in responses to water deficit in cassava. *Biol. Plant.* 58 469–478. 10.1007/s10535-014-0410-0

[B23] Rodríguez-Negrete E. A. Sánchez-Campos S. Cañizares M. C. Navas-Castillo J. Moriones E. Bejarano E. R. (2014). A sensitive method for the quantification of virion-sense and complementary-sense DNA strands of circular single-stranded DNA viruses. *Sci. Rep.* 4:6438. 10.1038/srep06438 25241765 PMC5377365

[B24] Romanel E. Silva T. F. Corrêa R. L. Farinelli L. Hawkins J. S. Schrago C. E. (2012). Global alteration of microRNAs and transposon-derived small RNAs in cotton (*Gossypium hirsutum*) during Cotton leafroll dwarf polerovirus (CLRDV) infection. *Plant Mol. Biol.* 80 443–460. 10.1007/s11103-012-9959-1 22987114

[B25] Schmittgen T. D. Livak K. J. (2008). Analyzing real-time PCR data by the comparative C(T) method. *Nat. Protoc.* 3 1101–1108. 10.1038/nprot.2008.73 18546601

[B26] Small I. D. Peeters N. (2000). The PPR motif- a TPR-related motif prevalent in plant organellar proteins. *Trends Biochem. Sci.* 25 46–47. 10.1016/s0968-0004(99)01520-0 10664580

[B27] Tang Y. Wang F. Zhao J. Xie K. Hong Y. Liu Y. (2010). Virus-based microRNA expression for gene functional analysis in plants. *Plant Physiol.* 153 632–641. 10.1104/pp.110.155796 20388670 PMC2879806

[B28] Tong A. Yuan Q. Wang S. Peng J. Lu Y. Zheng H. (2017). Altered accumulation of osa-miR171b contributes to *Rice stripe virus* infection by regulating disease symptoms. *J. Exp. Bot.* 68 4357–4367. 10.1093/jxb/erx230 28922766 PMC5853540

[B29] Voinnet O. (2005). Non-cell autonomous RNA silencing. *FEBS Lett.* 579 5858–5871. 10.1016/j.febslet 16242131

[B30] Wang B. Wang L. Chen F. Yang X. Ding M. Zhang Z. (2016). MicroRNA profiling of the whitefly *Bemisia tabaci* Middle East-Aisa Minor I following the acquisition of *Tomato yellow leaf curl China virus*. *Virol. J.* 13:20. 10.1186/s12985-016-0469-7 26837429 PMC4736103

[B31] Wang S. Cui W. Wu X. Yuan Q. Zhao J. Zheng H. (2018). Suppression of nbe-miR166h-p5 attenuates leaf yellowing symptoms of *Potato virus X* on *Nicotiana benthamiana* and reduces virus accumulation. *Mol. Plant Pathol.* 19 2384–2396. 10.1111/mpp.12717 30011130 PMC6638021

[B32] Wang S. T. Sun X. L. Hoshino Y. Yu Y. Jia B. Sun Z. W. (2014). MicroRNA319 positively regulates cold tolerance by targeting OsPCF6 and OsTCP21 in rice (*Oryza sativa* L.). *PLoS One* 9:e91357. 10.1371/journal.pone.0091357 24667308 PMC3965387

[B33] Wu J. Yang R. Yang Z. Yao S. Zhao S. Wang Y. (2017). ROS accumulation and antiviral defence control by microRNA528 in rice. *Nat. Plants* 3:16203. 10.1038/nplants 28059073

[B34] Wu J. Yang Z. Wang Y. Zheng L. Ye R. Ji Y. (2015). Viral-inducible Argonaute18 confers broad-spectrum virus resistance in rice by sequestering a host microRNA. *eLife* 4:e05733. 10.7554/eLife.05733 25688565 PMC4358150

[B35] Xia Z. Zhao Z. Gao X. Jiao Z. Wu Y. Zhou T. (2019). Characterization of maize miRNAs in response to synergistic infection of *Maize chlorotic mottle virus* and *Sugarcane mosaic virus*. *Int. J. Mol. Sci.* 20:3146. 10.3390/ijms20133146 31252649 PMC6650953

[B36] Xu D. Mou G. Wang K. Zhou G. (2014). MicroRNAs responding to *Southern rice black-streaked dwarf virus* infection and their target genes associated with symptom development in rice. *Virus Res.* 190 60–68. 10.1016/j.virusres 25038403

[B37] Xu X. Zhang Q. Hong J. Li Z. Zhang X. Zhou X. (2019). Cryo-EM structure of a Begomovirus geminate particle. *Int. J. Mol. Sci.* 20:1738. 10.3390/ijms20071738 30965627 PMC6480954

[B38] Yan F. Guo W. Wu G. Lu Y. Peng J. Zheng H. (2014). A virus-based miRNA suppression (VbMS) system for miRNA loss-of-function analysis in plants. *Biotechnol. J.* 9 702–708. 10.1002/biot.201300523 24664983

[B39] Yang J. Zhang F. Li J. Chen J. P. Zhang H. M. (2016). Integrative Analysis of the microRNAome and transcriptome illuminates the response of susceptible rice Plants to *Rice rtripe virus*. *PLoS One* 11:e0146946. 10.1371/journal.pone.0146946 26799317 PMC4723043

[B40] Zhang B. Wang Q. Pan X. (2007). MicroRNAs and their regulatory roles in animals and plants. *J. Cell Physiol.* 210 279–289. 10.1002/jcp.20869 17096367

[B41] Zhang C. Ding Z. Wu K. Yang L. Li Y. Yang Z. (2016). Suppression of jasmonic acid-mediated defense by viral-inducible microRNA319 facilitates virus infection in rice. *Mol. Plant.* 9 1302–1314. 10.1016/j.molp.2016.06.014 27381440

[B42] Zhou W. Cheng Y. Yap A. Chateigner-Boutin A. L. Delannoy E. Hammani K. (2009). The Arabidopsis gene YS1 encoding a DYW protein is required for editing of rpoB transcripts and the rapid development of chloroplasts during early growth. *Plant J.* 58 82–96. 10.1111/j.1365-313X.2008.03766.x 19054358

[B43] Zorzatto C. Machado J. P. Lopes K. V. Nascimento K. J. Pereira W. A. Brustolini O. J. (2015). NIK1-mediated translation suppression functions as a plant antiviral immunity mechanism. *Nature* 520 679–682. 10.1038/nature14171 25707794 PMC4779052

