# Supplementary material for: Tobacco curly shoot virus Down-Regulated the Expression of nbe-miR167b-3p to Facilitate Its Infection in Nicotiana benthamiana
Source: Front Microbiol. 2021 Dec 16;12:791561. doi: 10.3389/fmicb.2021.791561 (PMC8716884; doi:10.3389/fmicb.2021.791561)
Supplement: Supplementary file 1 [file Data_Sheet_1.DOCX]

GenBank. SubmissionID is SUB10511959 and BioProject ID is PRJNA770768
