# Supplementary material for: Tobacco curly shoot virus Down-Regulated the Expression of nbe-miR167b-3p to Facilitate Its Infection in Nicotiana benthamiana
Source: Front Microbiol. 2021 Dec 16;12:791561. doi: 10.3389/fmicb.2021.791561 (PMC8716884; doi:10.3389/fmicb.2021.791561)
Supplement: Supplementary file 2 [file Table_1.DOCX]

Table S1 The primers used in this paper

| Primer name | Primer sequence（5^，^-3^，^） |
| --- | --- |
| TbCSV-F | ATGCCTCAGCCAAGAAAACTTTT |
| TbCSV-R | TCAACACGACGACGTCTGTTCCC |
| miRNAOE-F | GGGATCCCAAACACACGCTCGGACGC |
| miRNAOE-R | GGAGCTCCATGGCGATGCCTTAAATAAAG |
| pCVA det-F | CTCATGTTCACGAGAATCATG |
| pCVA det-R | GCTATTAAGGCTAACTGAGTG |
| pCVBL1-F | ATGTATCCTACAAAGTTTAGGC |
| pCVBL1-R | TTAACCTAAATAATCAAGATCGT |
| PVX-det F | CGATCTCAAGCCACTCTCC |
| PVX-det R | GCTAGCTGGTGCTGACATC |
| TRV1-F | ATGGCGAACGGTAACTTCAAGTTG |
| TRV1-R | AGAACTTCATGAACAATCACTGG |
| TRV det-F | GGACGAGTGGACTTAGATTC |
| TRV det-R | CACGGATCTACTTAAAGAACC |
| PRCP(s)-F | CGAGCTCGATGAAGCTG CCAATGTTTT |
| PRCP(s)-R | CGGGATCCACCAAGCTCA ACTGATGCAA |
| miR167b-3p-RT | GTTGGCTCTGGTGCAGGGTCCGAGGTATTCGCAC CAGAGCCAACATTCAA |
| miR167b-3p-qF | GCGTATGAGGTCATCTAGCAGC |
| miRNA-qR | CTGGTGCAGGG TCCGAG GTA |
| PRCP-qF | ATGCATGCTTATGGTTGTAAGC |
| PRCP-qR | TTGTCTCGCGTCTTCAATTCTG |
| 35AV1-qF | CGCCGCCGTCTCAACTTCG |
| 35AV1-qR | GACTGGACCTTACATGGACCTTCAC |
| action-qPCR-F | CTTGAAACAGCAAAGACCAGC |
| action-qPCR-R | CATCCTATCAGCAATGCCCG |
