# Supplementary material for: Tobacco curly shoot virus Down-Regulated the Expression of nbe-miR167b-3p to Facilitate Its Infection in Nicotiana benthamiana
Source: Front Microbiol. 2021 Dec 16;12:791561. doi: 10.3389/fmicb.2021.791561 (PMC8716884; doi:10.3389/fmicb.2021.791561)
Supplement: Supplementary file 3 [file Table_2.DOCX]

Table S2 The phRobot predicted coding transcripts of nbe-miR167b-3p

| miRNA name | target geneID | Expectation | Inhibition | Target_Desc. |
| --- | --- | --- | --- | --- |
| nbe-miR167b-3p | Niben101Scf05269g02002.1 | 2.5 | Cleavage | sp\|Q9FRI5\|PPR57_ARATH *-*- Pentatricopeptide repeat-containing protein IPR002885 (Pentatricopeptide repeat), IPR011990 (Tetratricopeptide-like helical domain) GO:0005515 (protein binding) |
|  | Niben101Scf01167g02004.1 | 2.5 | Cleavage | sp\|Q9FXH1\|PPR52_ARATH *-*- Pentatricopeptide repeat-containing protein IPR002885 (Pentatricopeptide repeat), IPR011990 (Tetratricopeptide-like helical domain) GO:0005515 (protein binding) |
